# Climate change, fisheries management and fishing aptitude affecting spatial and temporal distributions of the Barents Sea cod fishery

**DOI:** 10.1007/s13280-017-0955-1

**Published:** 2017-10-26

**Authors:** Arne Eide

**Affiliations:** 1 Faculty of Biosciences, Fisheries and Economics, UiT – The Arctic University of Norway, Breivika, 9037 Tromsø, Norway; 2 NOFIMA, 9291 Tromsø, Norway

**Keywords:** Climate change, Fisheries economics, Fleet diversity, Spatial distribution

## Abstract

Climate change is expected to influence spatial and temporal distributions of fish stocks. The aim of this paper is to compare climate change impact on a fishery with other factors impacting the performance of fishing fleets. The fishery in question is the Northeast Arctic cod fishery, a well-documented fishery where data on spatial and temporal distributions are available. A cellular automata model is developed for the purpose of mimicking possible distributional patterns and different management alternatives are studied under varying assumptions on the fleets’ fishing aptitude. Fisheries management and fishing aptitude, also including technological development and local knowledge, turn out to have the greatest impact on the spatial distribution of the fishing effort, when comparing the IPCC’s SRES A1B scenario with repeated sequences of the current environmental situation over a period of 45 years. In both cases, the highest profits in the simulation period of 45 years are obtained at low exploitation levels and moderate fishing aptitude.

**Electronic supplementary material:**

The online version of this article (doi:10.1007/s13280-017-0955-1) contains supplementary material, which is available to authorised users.

## Introduction

It is difficult to predict future development of Arctic marine ecosystems and, even more so, how these are affected by human interactions. Immediate effects of such interactions are not only functions of the level and profile of the human activity but also of current state and dynamics of the natural system. Spatial and temporal distributions of prey and predator species vary, depending both on external drivers (e.g. climate and fisheries; Murawski 1993) and internal dynamics (e.g. spawning migrations; Rose 1993; Carvalho 1993). Long-term effects are by nature more difficult to predict than short-term perturbations, being functions of previous interactions and poorly known dynamics causing variations in spatial and temporal distributions of the system.

This describes the complexity of a marine ecosystem in its natural state, including the environmental variation which may occur within the natural sample space of the system (often referred to as natural variation). Climate change could cause system perturbations, redistributing some, or large, parts of the systems sample space. A dramatic change in the sample space of the system may be referred to as an ecosystem shift (Scheffer et al. 2001).

On the other hand, Arctic marine ecosystems are highly specialised to cope with significant environmental fluctuations, between seasons within years and annual variations. The resilience of the system may be regarded as the evolutionary solution of significant natural system variations, where only those species capable of adapting and coping in the long run have survived. This may suggest that system exposed to highly fluctuating environmental conditions, as the boreal marine ecosystems, is less vulnerable than others toward changes caused by climate change.

When looking at the exploitation of the cod (*Gadus morhua*) stock in the Barents Sea, the resilience of the Northern cod fishery is confirmed by archaeological fishbone analyses (Barrett et al. 2008, 2011) showing that dried cod continuously has been exported from the remote sub-Arctic region to other European countries over a period of more than thousand years. This period includes both the Medieval Warm Period (about 900–1400, Stocker et al. 2013), with significantly warmer climate than today, and the Little Ice Age (1450–1850, Stocker et al. 2013), which we temperature wise still are recovering from (Bianchi and McCave 1999).

While it may seem like a paradox that the fishery holding the longest documented trade history is found within the extreme naturally fluctuating environment in the sub-Arctic, the reasoning above indicates rather that the sub-Arctic is a place where we could expect to find resilient marine ecosystem. Both the human system as well as the marine ecosystems in this area are highly adapted to cope with extreme natural fluctuations.

This paper focuses the Northeast Arctic (NEA) cod fishery, the most important fishery in the Barents Sea. The NEA cod stock employs a variety of different coping strategies to adapt to a fluctuating physical and biological environment such as spawning and feeding migratory patterns, cannibalism, maturation dynamics and opportunistic feeding strategies (Sætersdal and Loeng 1987; Brown et al. 1989; Jørgensen et al. 2008; Kjesbu et al. 2014). The study employs a simulation model emphasising the migratory patterns (spawning and feeding migrations) constituting the most important spatial and temporal model variables. The aim of the study is to compare model results when assuming the current conditions to prevail (zero scenario) versus corresponding results under climate change conditions (climate change scenario).

Given the difficulties of fully understanding the system dynamics in its natural state, the difficulties of predicting the effects of a possible system perturbation caused by climate change become even more challenging. But more so, also observing the actual configurations of a marine ecosystem or mapping its recent history in all details is virtually impossible. The aim of this paper therefore is not to predict or forecast the NEA cod fishery under the two scenarios but rather to present possible outcomes within the sample spaces of the two scenarios (which certainly turn out to also have large overlapping areas, though not being the focus of this study). The climate scenario is based on the IPCC AR4 SRES A1B scenario (Anon. 2007) which at that time (2007) was considered being reasonably realistic. The A1 storyline assumes political focus on economics rather than environmental issues and a globalised economy. Among the different scenarios within the A1 family, the A1B scenario assumes a balanced development of energy technologies. The recent assessment report indicates that the A1B scenario may be too optimistic and less realistic than first anticipated (Stocker et al. 2013).

The focus on spatial distributions and fleet diversity is motivated from the widespread expectation that northern fish species will shift to a more northern distribution caused by increased water temperatures (Perry et al. 2005). The modelling approach utilised in this study has been developed and presented in two previous papers (Eide 2014, 2016). While the previous studies focused on the problems of identifying impacts climate change may have on the Barents Sea cod fishery, this paper provides a comparative study of a selected climate scenario and a zero scenario where no climate effects are considered.

## Materials and methods

### The model

**Fig. 1:**
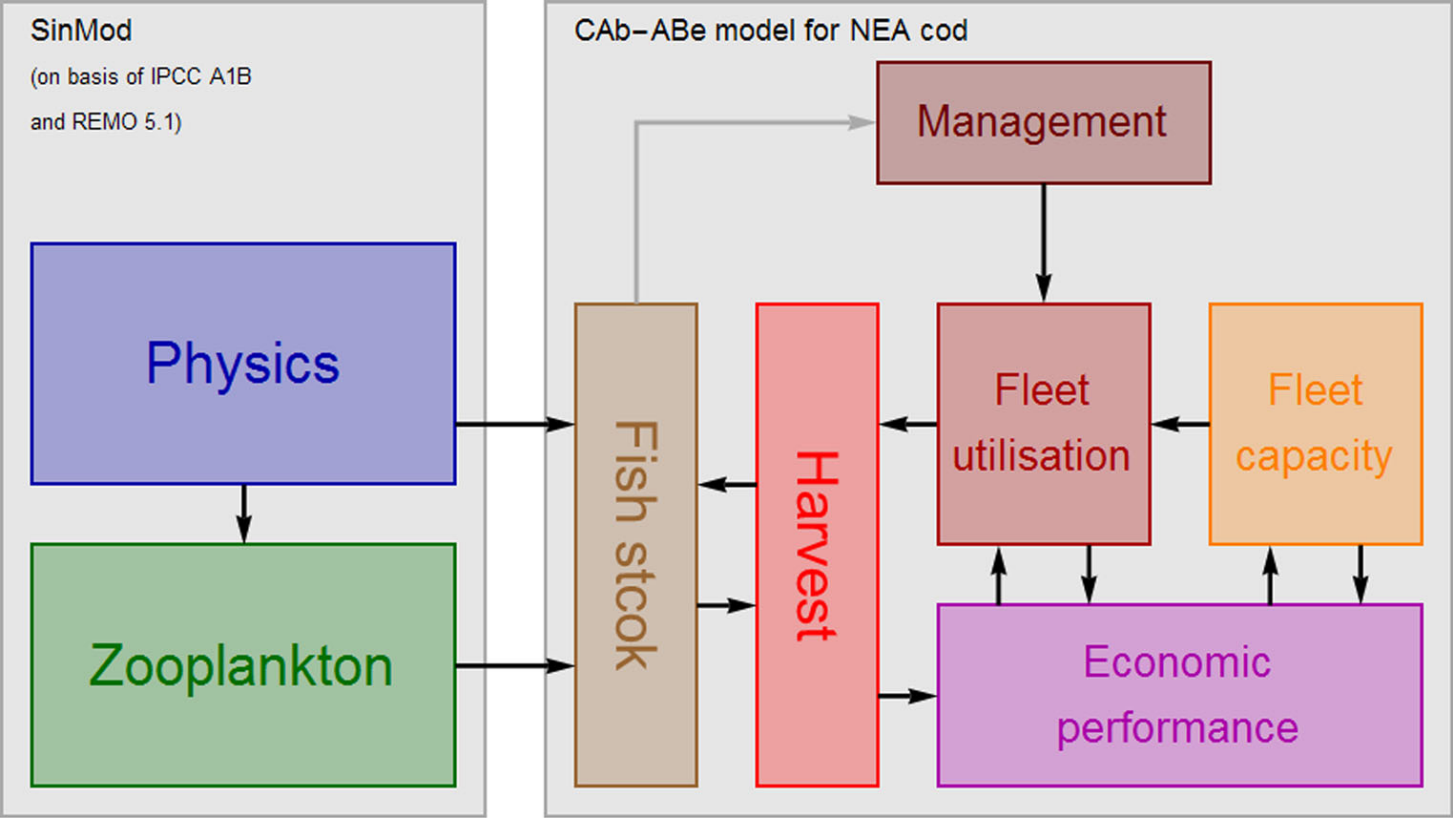
Model flow chart also indicating the one-way direction from the *SinMod* model to the *CAb*-*ABe* model. The automatised management module processes information about the state of the fish stock (the grey arrow) and set quotas based on given exploitation rates

The study makes use of a cellular automata model (*CAb*: Cellular Automata biological model) covering biological growth and spatial and temporal distribution of the cod stock. This model is run together with an agent-based model (*ABe*: Agent-Based economic model) defined within the same lattice, covering the economic exploitation of the stock. The flow chart of the combined *CAb*-*ABe*-model and the connected *SinMod* model is shown in Fig. 1. While the *SinMod* model (Slagstad et al. 2015) is a 3D model with a temporal resolution of 6 h (or less), the *CAb* module is a 2D spatial model with time unit 1 month.

*CAb* follows a normal set up with a uniform lattice of squared cells (80 × 80 km) with rules based on a Moore neighbourhood of range two. Each cell is defined in terms of geographical coordinates and the state variable of the cell is the cod biomass in the water column at the geographical position of the cell. Hence, the spatial distribution of the cod biomass at one point in time is given by the matrix of state variables in the lattice. According to the definition of Moore neighbourhoods (Hogeweg 1988) the rules are given as the percentwise distribution of the mid cell of a 5 × 5 cells matrix into all the 25 cells (at range = 2). With a time unit of 1 month, the cod distribution is recalculated monthly on basis of the current state variables, month-specific rules and the cell-specific growth properties. Biomass within each cell grows linearly towards the environmental carrying capacity level at which local stock collapse occurs so that only the fractional part of the biomass is left (while standardising the carrying capacity level to one). The natural mortality in the model is mainly covered by these local collapses, depending on monthly variation in carrying capacity levels and biomass levels in each cell after redistribution of biomass and biomass growth.

In Fig. 1, two arrows from *SinMod* point into the fish stock box in the *CAb*-*ABe*-module, representing the two datasets of monthly average ocean temperatures of each cell at 50-metre depth and the monthly biomass of small zooplankton species contained in each cell’s water column. In addition to these two datasets, *SinMod* also provides bathymetric data which by nature are fixed for the considered time period. The *SinMod* time series utilised in this study covers the 45-year period 2012–2057 aggregated to monthly intervals. *SinMod* data have in this study been converted from its original grid resolution of 20 km times 20 km to the *CAb*-*ABe* model resolution of 80 km times 80 km (see Eide 2014 for further details).

Information on spatial distribution of NEA cod for the period 2004–2010 has been provided through the *FishExChange* project^1^ by courtesy of the project staff. Catches in the database are registered on a quarterly basis while two surveys are carried out each year, *winter* survey (during April/May) and *ecosystem* survey (during August/September). Age-structured data from these data sources have been aggregated for the purpose of parameterising the *CAb*-*ABe* model. Registered catches and survey data have been spatially interpolated by Radial Basis Function interpolation (Myers 1994) followed by integration of the interpolated biomass surface. The integration has been performed over a geographic grid drawn as an equal size Lambert Azimuthal projection (corresponding to the projection used in the *SinMod* model with origin coordinates in 60°N, 58°E).

The data sample from the period 2004 to 2010 was considered to represent the current environmental situation, rather than reflecting ongoing changes in climate. There are several reasons for this. The period is rather short and the datasets, although displaying significant variations in the distributional patterns, do not show any significant trends or systematic changes. The seasonal variations are extreme but the seasonal biomass centres of gravity are almost identical each year during the period. In terms of weighted biomass distances for each quarter during the time period from a given geographical point (in the calculations the coordinates of Tromsø was chosen), cluster analyses did not reveal any systematic changes and different years constituted the main cluster for each of the four quarters.

**Fig. 2:**
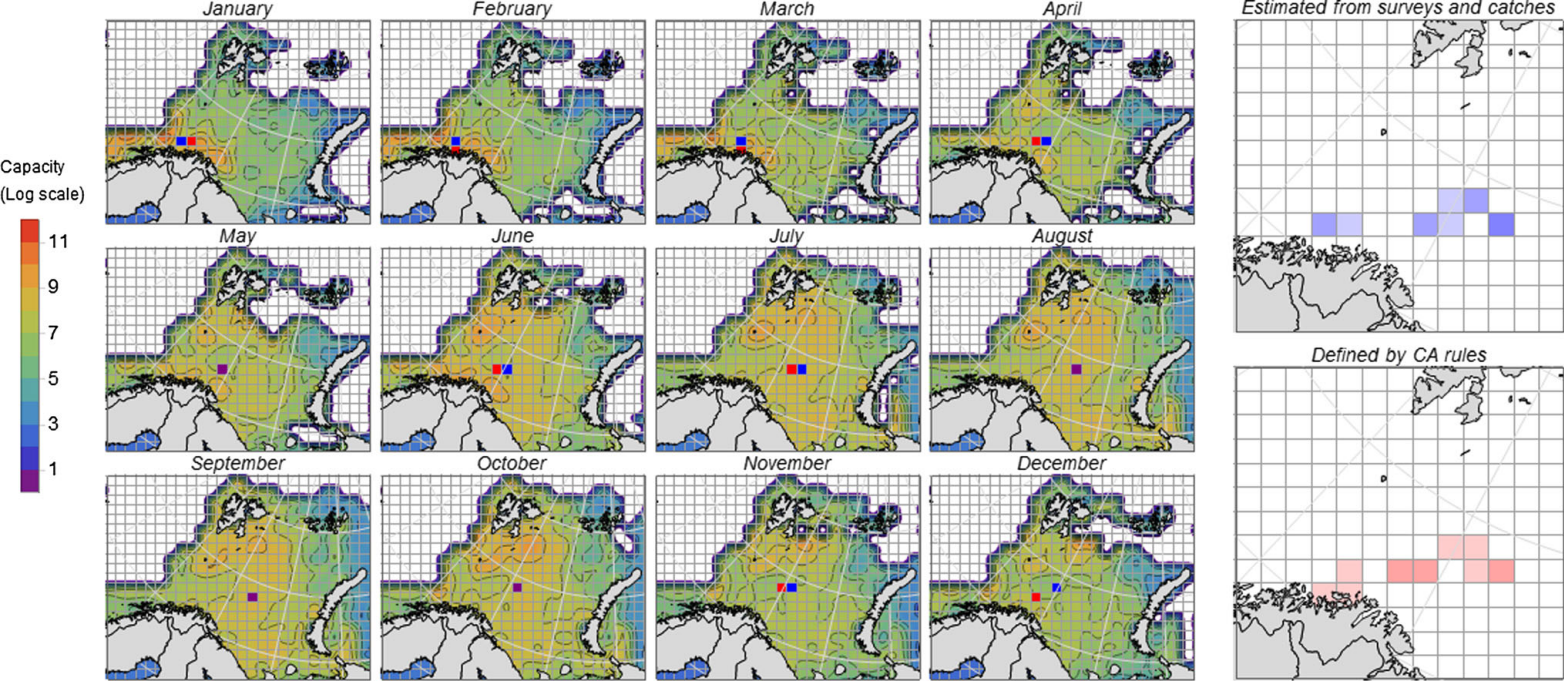
Monthly NEA cod distribution charts and cells of gravitation centres of biomasses, blue cells from the integrated biomass data from 2004 to 2010 and red cells corresponding model outputs. The two charts to the right provide the annual sample of monthly gravitation centres for the empirical observations (blue) and the model representation

Based on this, the data sample was considered as a representative distribution related to the current climatic conditions. The average monthly spatial distributions of NEA cod stock biomasses during the period 2004–2010 were found by merging the different sources of information relevant for each month as explained in Eide (2014). Resulting distributional maps for each month are shown in Fig. 2. All modifications made on the raw data received from *SinMod* and *FishExchanges* are made publicly available through UiT Open Research Data.^2^

Spatial and temporal distributions of the NEA cod environmental carrying capacity levels for each scenario have been estimated on the basis of constraining physical and biological factors in addition to the observed distributional patterns (Fig. 2). The NEA cod distribution as assumed to be constrained to ocean depths less than thousand metres and ocean temperatures higher than −1.5 °C (the monthly average at 50 m depths) (Eide 2014). In addition, a cell’s environmental carrying capacity is reduced by 80 % when small zooplankton densities fall below 2 g carbon per square metre, considering the density of small zooplankton being a proxy for food availability in the area.

Monthly estimated current carrying capacities are then modified according to *SinMod* datasets of bathymetry, temperatures and zooplankton biomasses over the simulation period, representing the changes in environmental carrying capacities corresponding to the A1B scenario. As stochastic element is added to estimated environmental carrying capacities. As the mean deviation of the carrying capacity of each cell varies between 20 and 30 % (following the seasonal pattern of cod availability) during the period of observations (2004–2010), a normally distributed stochastic element with a mean value of one and a standard deviation of 10 % is assumed. The stochastic element also serves to establish the zero scenario monthly carrying capacities, repeating the current climate with the minor perturbations caused by the stochastic process.

**Fig. 3:**
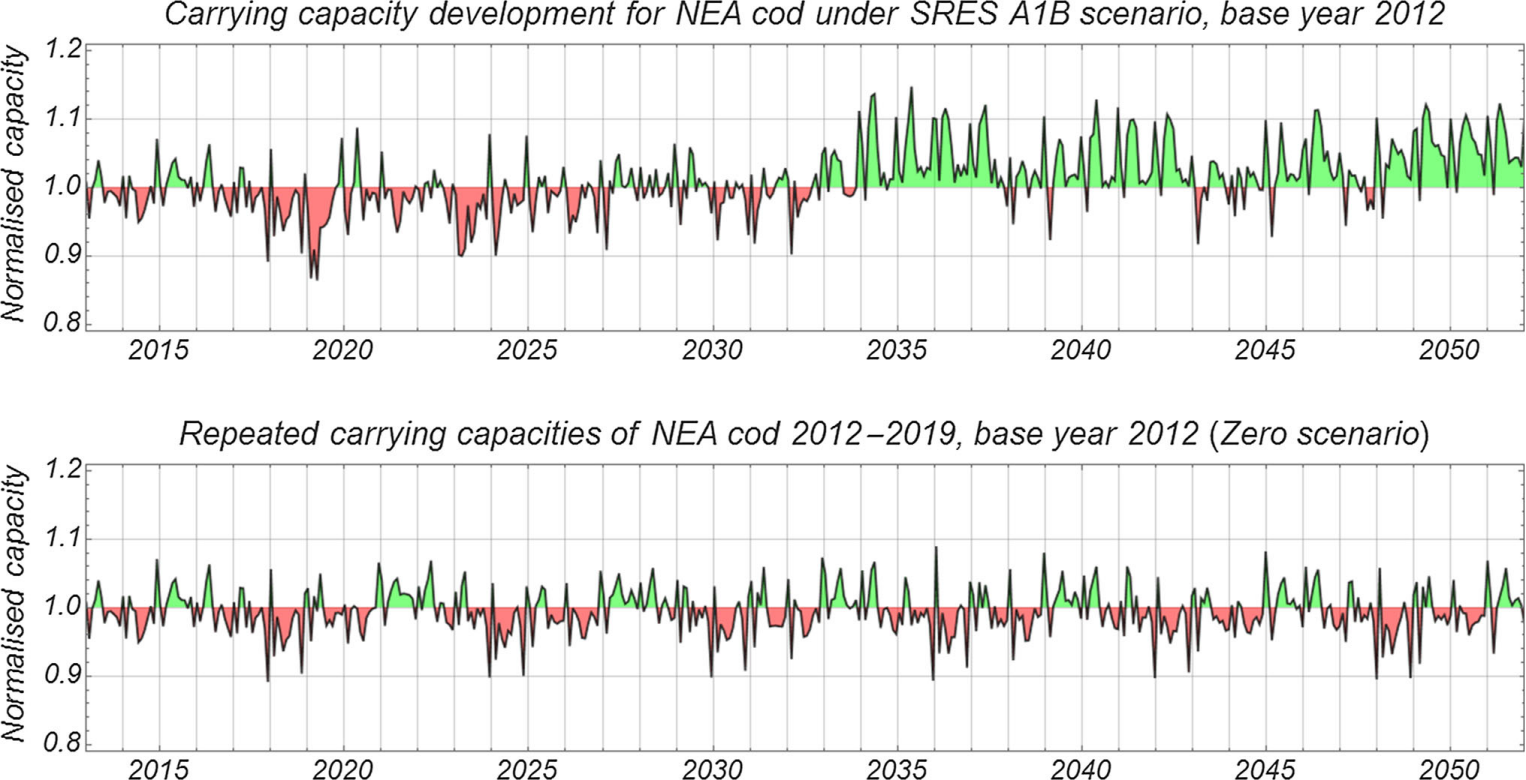
Monthly aggregates of normalised (base year 2012) carrying capacities for NEA cod based on initial distribution data from the *FishExChange* project (2004–2010). The upper panel shows the carrying capacity development over the period when utilising data from the *SinMod* A1B simulations while the lower panel shows the corresponding zero scenario, repeating the environmental conditions of the first 6 years throughout the simulation period

Figure 3 displays the total NEA cod environmental carrying capacity anomalies of the two scenarios throughout the simulation period. The A1B scenario is essentially as presented in Eide (2014) while the zero scenario is defined as repeated sequences of the first 6 years of the A1B scenario which is presented in Eide (2014, 2016). Both the zero and the A1B scenario show ±10 % fluctuations related to the base year (2012), while the A1B scenario (upper panel in Fig. 3) in the mid 2030s displays a shift upwards, resulting in almost a 10 % increase in carrying capacity compared with the base year.

When having established the cellular automata lattice with cell-specific carrying capacities, which develop according to environmental variables and observed distributional pattern in the cod population, the next step is to establish cellular automata distributional rules. Essentially, the rules describe how individual cod moves in terms of directions and distances within the time frame of 1 month. According to Rose et al. (1995), NEA cod may have a range between 210 and 720 km over a period of 30 days, indicating that three cells in all direction from a given cell in a 80 × 80 km grid represent a reasonable range (range = 2, assuming Moore neighbourhood).

The rules should in principle be able to move the cod biomasses over time according to previous observations. This boils down to a straight forward statistical problem minimising the sum of squared distances between the observed centres of gravity in the observed cod biomass and the centres of gravity in the by rule distributed cod biomass (described in detail in Eide 2016). The best model fit is indicated by the red cells in Fig. 2, while the observed centres of gravity (based on surveys and catch information) are shown as blue cells in the same figure. The minimised sum of squares of the 12 observations equals 6.62 (measured in square units) within a distribution of monthly centres of gravity spanning over 8 (horizontally) times 2 (vertically) cells (Eide 2014). This means that the rules perform sufficiently well in replicating observed migratory pattern in the NEA cod stock. The rules are month specific and identical for all cells for each month.

The shifting carrying capacity distributions constitute the model environment and mimic the changes both in the physical and biological environment in which the cod stock lives, defining rich areas allowing the cod stock to expand and poor areas in which saturation levels are reached at low biomass levels. By affecting the distribution of biomasses also the migration pattern is affected, even though the cellular automata distribution rules are fixed for the whole simulation period (Eide 2014).

**Fig. 4:**
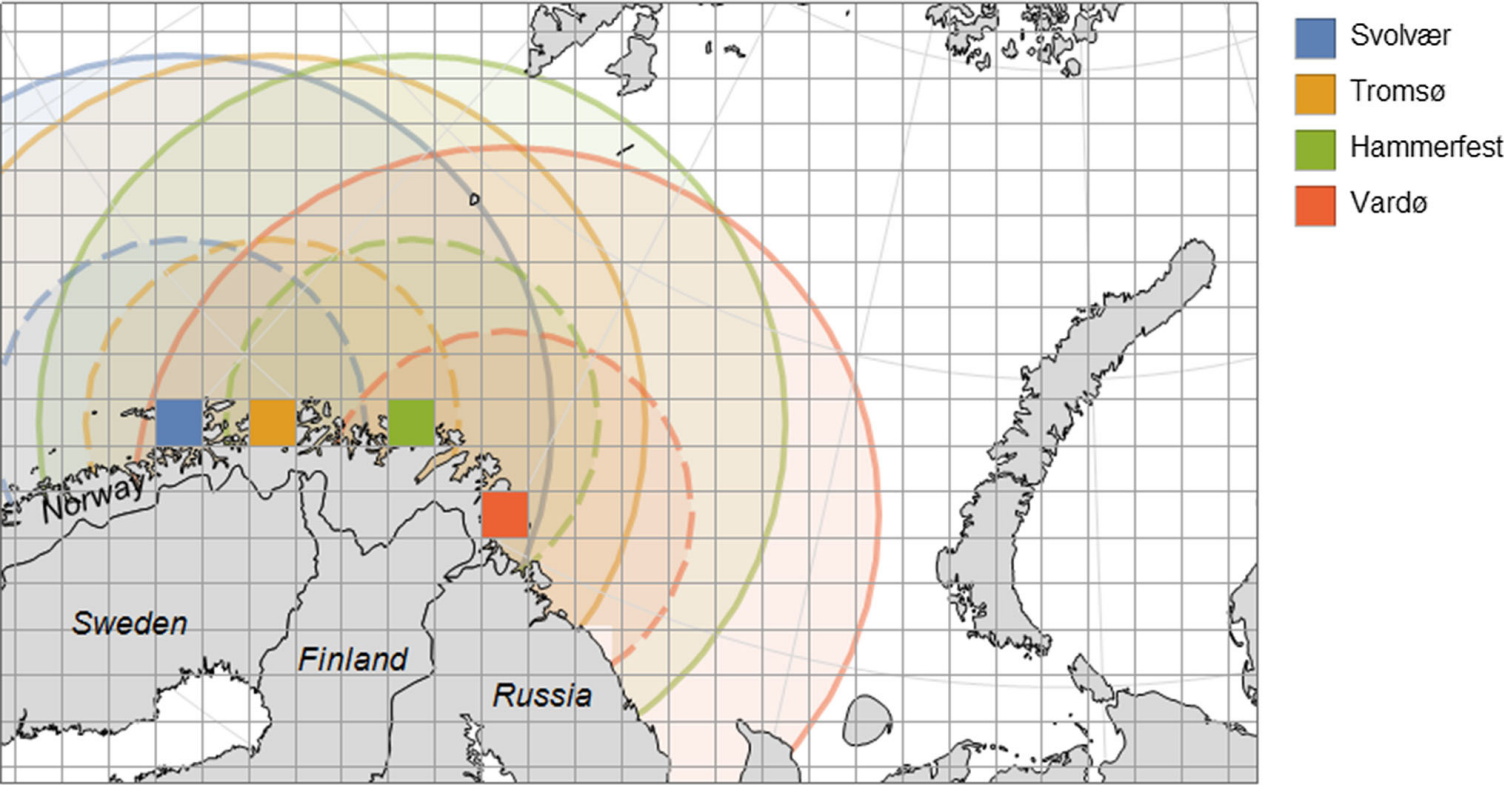
The map illustrates the geographical areas covered by each of the eight fleet in the model. The ranges of the high sea vessels are indicated by solid circles while ranges of the small-scale coastal vessels are indicated with dashed circles. The two vessel types are placed in four different ports along the North-Norwegian coast (Svolvær, Tromsø, Hammerfest and Vardø)

The *ABe* model includes four North-Norwegian fishing ports (Svolvær, Tromsø, Hammerfest and Vardø) and two fleet types (small and large vessels) placed in each of these ports (Fig. 4). The small vessels represent coastal fishing vessels with an assumed monthly range of four cells, while the large vessels may operate in the high sea, having a monthly range of eight cells.

Hannesson (1983) and Eide et al. (2003) suggest that the stock-output elasticities in harvest production differ significantly between fleet groups in the NEA cod fishery. In order to accommodate different stock-output elasticities for coastal and high sea fishing vessels, a Cobb–Douglas product equation is used to express the monthly fleet harvest ($h_{i}$) in cell *i* when fishing effort is $e_{i}$ and stock biomass $x_{i}$,

1 $$ h_{i} \left( {e_{i} ,x_{i} } \right) = q e_{i}  x_{i}^{\beta } , $$

where *q* is the catchability coefficient and *β* is the stock-output elasticity of the fleet, $0 \le \beta \le 1$.

Similarly to Heen and Flaaten (2007), Hannesson (1975), and Eide (2007, 2008, 2016), we assume the cod fleets to be price takers. Following this approach, this study assumes a fixed price (*p*) per unit of harvest. The fleet revenue (*re*) obtained in cell *i* is

2 $$ re_{i} \left( {e_{i} ,x_{i} } \right) = p  h_{i} \left( {e_{i} ,x_{i} } \right) $$

and corresponding variable cost (*vc*) of the fishing operation is

3 $$ vc_{i} (e_{i} ,d_{i} ) = (c_{\text{e}} + c_{\text{d}} d_{i} )  e_{i}, $$

where the variable $d_{i}$ is the distance from homeport to cell *i.* $c_{\text{e}}$ and $c_{\text{d}}$ are post parameters, unit cost of effort and per unit of effort unit cost of distance, respectively. Apart from being operated from four different ports (causing differences in variable costs due to different distances to home ports), each of the two types of vessels (small-scale and high sea vessels) is assumed to be homogeneous in terms of technology and economy. However, the two types of vessels differ from each other in both of these dimensions.

The fleet contribution margin of all cells are found by Eqs. (2) and (3) when summing revenues and cost for all cells. Negative contribution margin will cause the fleet not to fish since the revenue is not sufficient to cover running cost. After adjusting fishing effort accordingly, total annual fleet contribution margin (*cm*) of all cells is

4 $$ cm\left( {\varvec{e}, \varvec{x}} \right) = \mathop \sum \limits_{m = 1}^{12} \mathop \sum \limits_{i = 1}^{n} \left\{ {re_{m,i} \left( {e_{m,i} ,x_{m,i} } \right) - vc_{m,i} (e_{m,i} ,d_{i} )} \right\}. $$

The matrices ***e*** and ***x*** give the fishing effort of the fleet and stock biomasses distributed on cells and months. Index *m* indicates month number and *n* is the total number of cells available for the given fleet. Number of available cells depends both on the physical range of the vessel (Fig. 4) and the regulatory divisions of sea areas. In Norway, the high sea vessels are not allowed to fish inside four nautical miles from the baseline.

Annual profit is found by withdrawing the fixed cost (fc) from the contribution margin described in Eq. (4):

5 $$ \pi \left( {\varvec{e}, \varvec{x}} \right) = cm\left( {\varvec{e}, \varvec{x}} \right) - {\text{fc}}. $$

Total fleet fishing effort at time *t* (a given month in a given year) is the sum of the fishing effort distributed on all available cells:

6 $$ E_{t} = \mathop \sum \limits_{i = 1}^{n} e_{i,t}. $$

The fleet capacity in terms of maximum fishing effort which may be produced during a single month is *V*. The relation between absolute fleet size, *V*, and utilised fishing effort, *E*, is

7 $$ 0 \le E_{t} \le V_{t}. $$

This study assumes a pure or quota-regulated, open access fishery. Entry to and exit from the fishery are driven by profits beyond the normal level or negative profits, respectively. While Vernon Smith in his seminal paper (Smith 1968) assumed flow of capital into a fishery to be proportional to profit, this study assumes fixed entry and exit rates of vessels. The varying degree of fleet utilisation (*E/V*) may, however, bring the resulting dynamics closer to the dynamics assumed by Smith, since also fleet utilisation varies in space and time (e.g. negative contribution margins keep vessels in harbour). After introducing the entry (fg) and exit (fd) rates, the fleet dynamics are given by

8 $$ \begin{array}{*{20}c} {{\text{If}}\quad \pi_{t} \left( {\varvec{e}, \varvec{x}} \right) < 0\quad {\text{then}}\quad V_{t + 1} = (1 - {\text{fd}})V_{t} } \\ {{\text{If}}\quad \pi_{t} \left( {\varvec{e}, \varvec{x}} \right) > 0\quad {\text{then}}\quad V_{t + 1} = (1 + {\text{fg}})V_{t} }. \\ \end{array} $$

Entry rates are often expected to be higher than exit rates as in Eide (2007).

A reasonable assumption is that the fishers attempt to maximise their economic performance by fishing at the most profitable areas (e.g. within the most profitable cells). The problem is however to identify where the most profitable cells are positioned. The fishers search to solve this problem through the use of their best knowledge, experience and skills, including the use of fish finding technology, the information that may be obtained within the fishing community and from other sources, attitude towards risk and the economic factors constraining their activity. How successful the fishers are in identifying the most profitable areas depends in this model on the value of a single parameter, the smartness parameter *s*. The core expression for each vessel group in the model is given by

9 $$ e_{j,t} = \frac{{\left( {\frac{{re_{j,t} }}{{vc_{j,t} }}} \right)^{s} }}{{\mathop \sum \nolimits_{i = 1}^{n} \left( {\frac{{re_{i,t} }}{{vc_{i,t} }}} \right)^{s} }}  E_{t}, $$

where the distribution of fishing effort is determined by the ratios of Eqs. (2) and (3) and the value of smartness parameter *s*, reflecting the fleets aptitude of identifying the most profitable (in terms of the revenue/cost ratio) fishing grounds. The smartness parameter (*s*) is a lump-based parameter where a number of features are reduced down to the value of this single parameter. The two extremes (*s* = 0 and *s* = ∞) go from a uniform distribution of fishing activities in the area available for the fleet (*s* = 0, representing total ignorance) to placing all fishing activities into one single cell (*s* = ∞, perfect knowledge). For the special situation s = 1, the distribution of fishing activities exactly follows the distribution of profit opportunities (expressed by revenue/cost ratios).

In the following, *s* = 1 is regarded to be the lowest smartness level of interest, while a possibly unrealistically high level of *s* = 10 is the highest smartness level included in the study. The range $s \in \{ 1, 10\}$, which spans out a large variety of distributional patterns and the range are considered to cover actual levels of knowledge and insight in possible distributional patterns forming the base of rational decisions on where to fish. A smartness parameter value equal one clearly is far below the expected smartness levels of today’s fisheries, while a smartness value equal 10 appears to be too optimistic with respect of level of insight and fishing aptitude. A qualified guess is that the most realistic smartness value is somewhere in the range of 2–3, depending on individual experience, knowledge, technical measures as well as social factors. In this study, a global smartness value is assumed to be global within each simulation. The model parameter setting is presented in Table 1.

**Table 1:** Values used for fleet parameters and variables between model simulations [from Eide (2016)]

Parameters and variables	Symbol	Small vessels	Large vessels
Unit price of harvest (NOK/kg)	*p*	13.00	13.00
Stock-output elasticity	*β*	0.70	0.50
Catchability coefficient	*q*	0.66	0.24
Unit cost of effort (mill. NOK/standardised effort)	*c*_e_	0.00035	0.00055
Unit cost of distance (mill. NOK/80 km)	*c*_d_	0.00025	0.00035
Fixed cost per year (mill. NOK/year)	fc	30	60
Annual fleet entry rate (%)	fg	10	7
Annual fleet exit rate (%)	fd	8	5
Monthly fleet range (cells, 80 × 80 km)	–	4	8
Quota share (%)	–	60	40
Fishing effort^a^	*E*	≤ *V*	
Fleet capacity (in terms of possible fishing effort)^a^	*V*	≥ *E*	
Smartness coefficient^b^	*s*	1–10	
Fishing mortality rate used in quota setting^b^	*F*	0.1–0.4	

^a^Dynamic variable within simulations

^b^Variable between simulations

The study includes different governmental constraints represented by four different management regimes of which one is no management (open access). The other three management regimes are all in principle structured similarly to the current management system, assuming different exploitation rates and perfect management control. A total allowable catch (TAC) is set according to a given target levels of the fishing mortality rate (*F*), assuming perfect stock information.

The NEA cod stock is equally shared between Norway and Russia, and a Russian catch of the same quantity as the Norwegian catch is included without defining specifically a Russian fleet. The Russian capture is assumed to be high sea catches following the distribution of cod biomasses in areas available for Russian vessels.

The total Norwegian quota is shared between coastal (small) vessels and high sea (large) vessel in a fixed ratio (60/40), which is a slight simplification of the current quota allocation system. The high sea vessels are not allowed to fish inside four nautical miles from the baseline, which is implemented by limited access (25 % of total area) to cells along the coast.

This study investigates and compares distribution and variability in the two scenarios, in particular emphasising fleet diversity and spatial distribution of the fishing activity. While previous studies (Eide 2007, 2008) suggest that fisheries management may have a greater impact than climate change on the biological development and economic performance of Arctic groundfish fisheries, these studies did however not include spatial distributions of biomasses and fishing effort.

## Results and discussion

Twenty-four simulations within each scenario were performed, each scenario combining six smartness levels (represented by the *s*-values 1, 1.5, 2, 3, 5 and 10) and four management regimes (*F* = 0.1, *F* = 0.2, *F* = 0.4 and open access). The fleet dynamics is in all simulations controlled as described above, inducing a total fleet capacity (number of vessels that may participate in the fishery, *V*) which may be larger or equal to the active fleet *E*: $V \ge E$. Over time, the fleet size (*V*) and the fishing effort (*E*) follow different paths in different fishing ports and for the two types of fleets. The Shannon function *H* is used as a fleet diversity index (Eide 2016), mapping how fleet diversity develops in the different simulations.

**Fig. 5:**
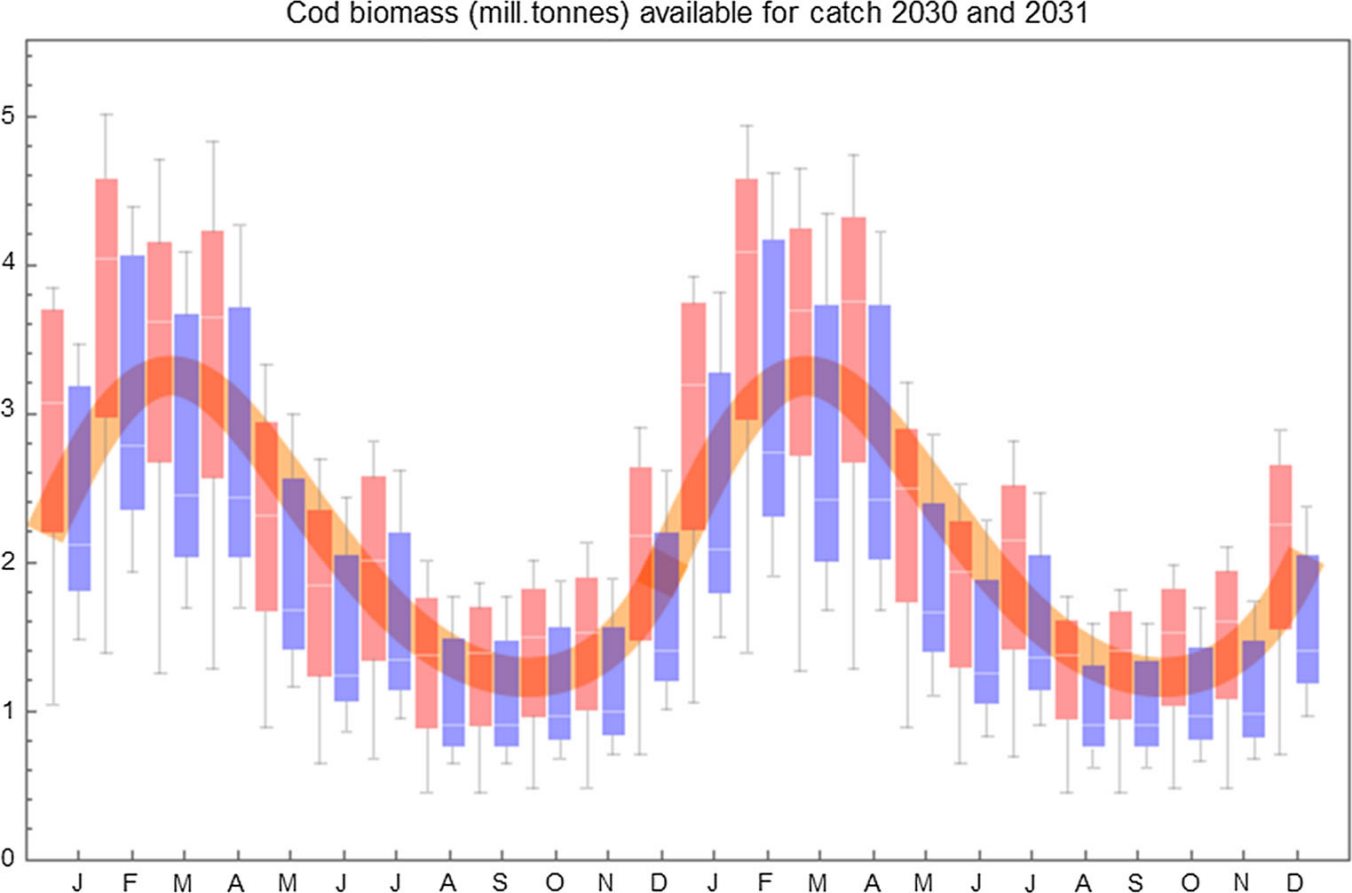
The Box–Whisker chart gives monthly values and variations over a period of 2 years (2030–2031) in the *CAb*-*ABe* model for all simulations, separated on the zero scenario (blue) and the A1B scenario (red). The thick, yellow curve is the catchability function found for the trawl fishery on the NEA cod stock in Eide et al. (2003)

Figure 5 shows monthly samples of biomass distribution outputs for all simulations over a period of 2 years (2030 and 2031), indicating how both scenarios follow the seasonal pattern in the cod stock available for exploitation. The expected season profile is displayed as a thick yellow curve, drawn from the mathematical expression for the season profile found in Eide et al. (2003). The variations indicated by each Box–Whisker item show the monthly variation within the 24 simulations performed within each of the two scenarios where the blue bars represent the zero scenario and the red bars the A1B scenario.

Even though the 2 years captured in Fig. 5 is just prior to the occurrence of a striking shift in the development in the A1B carrying capacity anomaly (as seen in Fig. 3 this happened about 2034), the A1B scenario biomasses shown in Fig. 5 are significantly higher than the corresponding biomasses representing the zero scenario. To a large degree, however, the two scenarios overlap each other and both describe seasonal paths in close accordance with the expected seasonal profile.

**Fig. 6:**
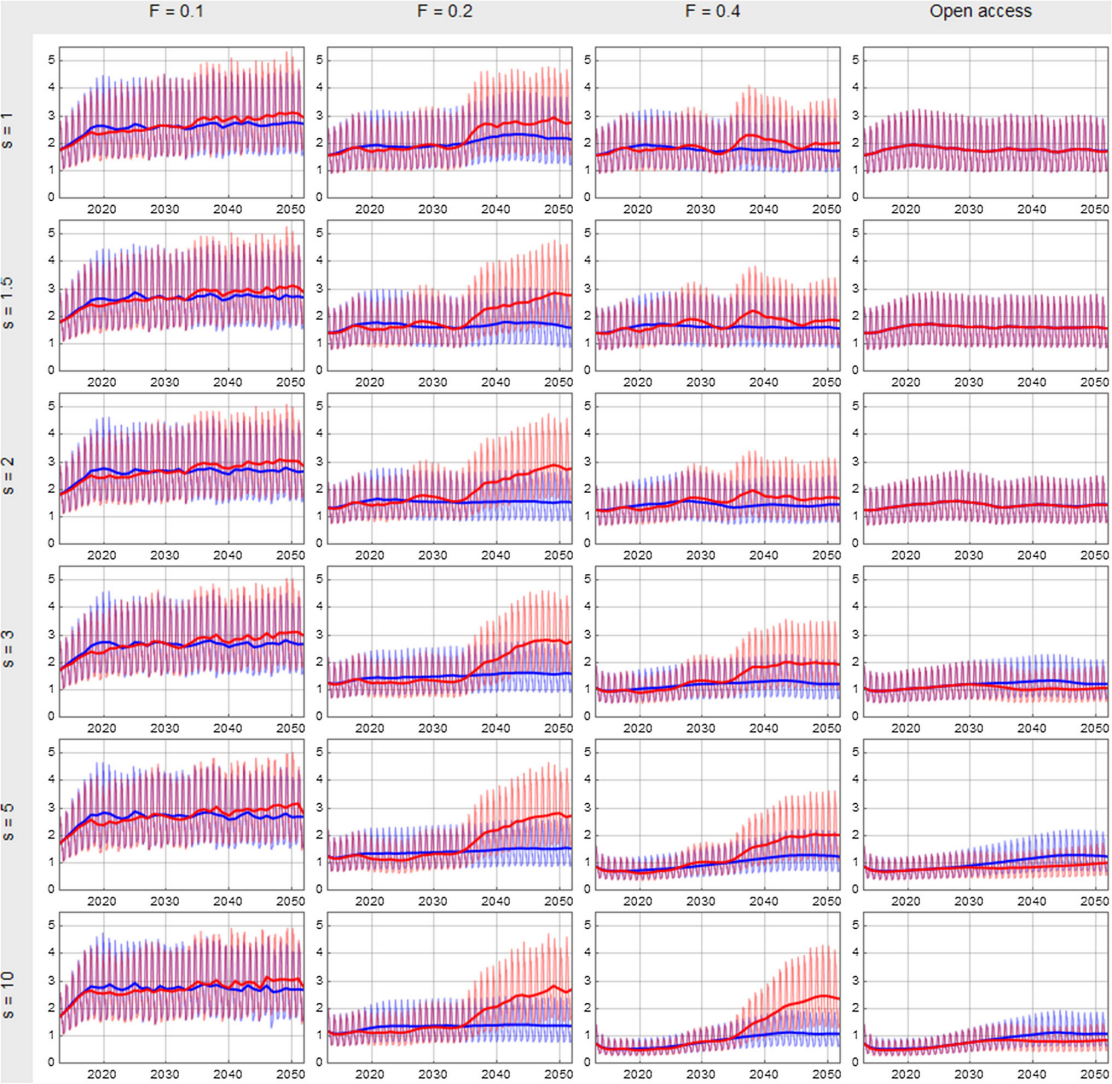
Total monthly NEA cod biomasses available for fishing (by the modelled fleets) from 2013 to 2052 for different combinations of the smartness parameter *s* and the exploitation rate. The thick solid curves give the annual averages while the thin curves connect the monthly biomasses. The blue colour represents the zero scenario and the red colour the A1B scenario. The vertical axes give the stock biomass in million tons

**Fig. 7:**
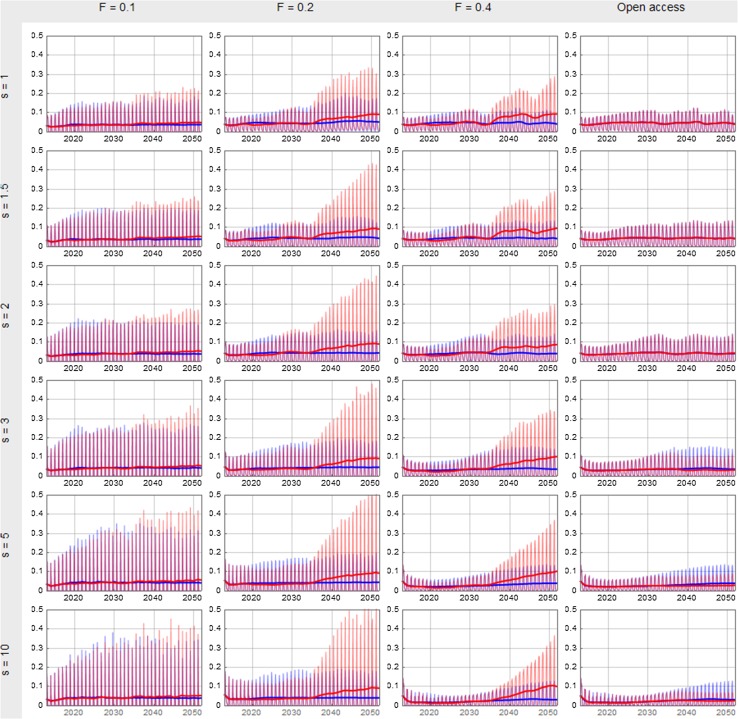
Monthly NEA cod total catches from 2013 to 2052 for different combinations of the smartness parameter *s* and the exploitation rate. The thick solid curves give the average monthly catches while the thin curves connect the actual monthly catches. The blue colour represents the zero scenario and the red colour the A1B scenario. The vertical axes give the catches in million tons

The shift suggested to occur around 2034 is also visible in Figs. 6 and 7, showing the biomass and catch developments for all the simulations. These figures unmask several interesting features. The seasonal profiles of the two scenarios follow to a large degree the same pattern up to the mid-thirties after which a significantly higher stock biomass appears in the cases of an exploitation rate based on a fishing mortality rate (*F*) equal 0.2 and 0.4. This effect is however not apparent in the case of *F* = 0.1 in which available stock biomass already is stabilised on a quite high level (around 3 million tons according to Fig. 6) in both scenarios. The effect of the shift in environmental carrying capacity is however reflected in increased monthly catches also in the case of *F* = 0.1, though significantly less than the increases seen in the cases of *F* = 0.2 and *F* = 0.4 (Fig. 7).

The unregulated fishery differs from the other three in Figs. 6 and 7, particularly after the shift in carrying capacity where stock biomasses, catches and seasonal peaks clearly are lower in open access fishery. At high smartness levels and open access fishery, monthly available biomasses and obtained catches in peak season even reach higher levels in the zero scenario than that in the A1B scenario.

The years after the environmental shift in the mid-thirties provides however the fleets with considerably higher profits in the A1B scenario than what is obtained in the zero scenario (Fig. S1 of the Electronic Supplementary Material). When comparing the profit surfaces of the two scenarios for all the years (top left in Fig. S1) with the last 25 years of the simulation (top right in Fig. S1 of the Electronic Supplementary Material), it becomes visible how the environmental effect contributes in lifting the whole profit surface of the A1B scenario.

Open access fishery combined with high smartness levels results in higher profits in the zero scenario than in the A1B scenario throughout the simulation period. At lower smartness levels, however, the profit surface area within the open access area reaches surprisingly high levels as seen in the lower-right table in Fig. S1, where the profit obtained the last 25 years in open access when *s* = 2 is close to the maximum overall profit (at *F* = 0.2 and *s* = 5). In general, the A1B scenario seems to give relatively larger benefits to higher smartness levels than the zero scenario does. In both cases, highest profits are found at the fishing mortality rate (*F*) 0.2 but while the smartness level in A1B scenario maximum is 5 it is 1.5 in the zero scenario.

Figure S1 also indicates, for both scenarios, that the largest profits are obtained at moderate levels of the smartness parameter s, in the range 1–3 in the zero scenario and 1–5 in the A1B scenario. The exception is for the last 25 years of the simulation period, when also higher exploitation levels contribute in large profits in the A1B scenario.

**Fig. 8:**
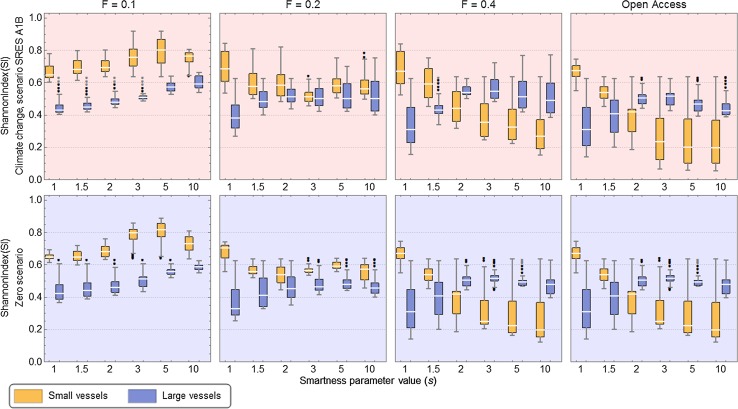
Fleet diversity indexes (based on the Shannon Function H, see Eide 2016) found for the zero scenario (below) and the A1B scenario (above) for the different management regimes and varying smartness (*s*) values

Eide (2016) introduces a fleet diversity index based on the Shannon Function H (Spellerberg and Fedor 2003) which is utilised in Fig. 8 and Fig. S2 of the Electronic Supplementary Material, differing between vessels belonging to the coastal and high sea fishing fleets. As higher values of the diversity index indicate higher diversity, clearly the coastal fleet exhibits the highest diversity at low exploitation levels and for low smartness values at all levels of exploitation. The two scenarios follow the same pattern in this respect and also regarding the trends while increasing smartness levels. As higher smartness levels seem to contribute in increased fleet diversity for the high sea vessels (for exploitation rates at *F* = 0.2 and above), the opposite is the case for the coastal fleet, though not so pronounced at *F* = 0.2 as for the highest exploitation levels. Although the fleet diversity for high smartness levels and open access seems to drop below the corresponding levels of the zero scenario and the opposite for *F* = 0.4, the general impression is that the fleet diversity in the A1B scenario corresponds very closely to the fleet diversities found in the zero scenario simulations.

The declining diversity for small-scale vessels at higher smartness values and exploitation rates is also clearly visible in Fig. S2. Each graphical plot in Fig. S2 is divided by the diagonal into two sector where the upper sector is the area where the high sea fleet exhibits a higher diversity than the coastal fleet, while it is opposite in the sector below. In Fig. S2, for the zero scenario as well as for the A1B scenario, at high exploitation rates (open access and when *F* = 04) and for s-values higher than 1.5, all points lay above the diagonal and hence indicate that the high sea fleet is more diverse than the coastal fleet.

The fact that the high sea fleet has a wider range than the coastal fleet may be the simple explanation of the higher diversity at high levels of exploitation. The advantage of a higher mobility combined with a minimum level of fishing aptitude becomes relatively a more important advantage as the exploitation level increases. As seen in Fig. 8, however, also the diversity of the high sea fleet may decrease at sufficiently high smartness levels when the exploitation level is high, while the decline in fleet diversity occurs at lower exploitation levels in the coastal fleet. It should however be noted that this picture may be completely reversed when including alternative fisheries which first of all provide the coastal fleet with different options that could contribute to a higher fleet diversity.

At low s-values in open access, the fleet diversities of the two scenarios are virtually identical. Overall, the highest fleet diversities are found at low exploitation levels and high smartness levels. The fleet diversities of the two scenarios follow each other closely but from the zero to the A1B scenario, the tendency is increasing diversity in the coastal fleet at low smartness while it is the high sea vessel diversity which increases at high smartness levels.

Figure S3 of the Electronic Supplementary Material shows the vertical and horizontal distributional ranges of the gravity centres of stock biomass and fishing effort distributions over the 45 simulated years. The stock distribution in terms of centres of gravity turns out to be very stable, almost not affected of fishing intensity and levels of smartness. A slight North-eastern movement is indicated for the A1B scenario compared with the zero scenario but the main impression is that the stock biomass distribution does not change. In the case of open access, the two scenarios are practically equal in terms of stock biomass distribution.

Significantly larger changes are seen in the distribution of fishing effort, also reflecting the changing fleet compositions due to stock properties, exploitation levels and smartness. At increasing levels of smartness, there is a light tendency towards a more South-western distribution of fishing effort in both scenarios, even for the A1B scenario where the stock distribution slightly moves in the opposite direction. This indicates that the effect of reducing cost related to distance from port may be a more important factor than the possibly more North-eastern stock distribution.

Some details of the information embedded in Fig. S3 come out in Fig. S4 of the Electronic Supplementary Material, showing how the stock biomass distribution clusters for the two scenarios and their different combinations of fishing intensity and smartness levels. The two scenarios come out as independent clusters for all smartness levels at the lowest exploitation rate (*F* = 0.1), while a more mixed picture is seen at higher exploitation levels. At the higher exploitation levels, the differences between the different scenarios and combinations are smaller but still a distinct clustering between scenarios are visible.

This is however not the case for the distribution of catch and effort (Fig. S5 and S6 of the Electronic Supplementary Material), which for natural reasons are closely related. In these cases, the lowest exploitation level and low smartness levels cluster independent of climate scenario. It seems also to be a combined clustering tendency for both scenarios at higher exploitation levels and higher smartness levels, suggesting that the distribution of effort and hence catches is more depending on smartness levels and fishing intensities than marginal changes in the distribution of stock biomasses.

## Conclusion

The idea of the NEA cod moving into a more northern distribution area is not supported by the findings of this study. On the contrary, the centres of gravity of the cod biomass distribution are surprisingly stable throughout the simulation period. While the distribution area in north, south and west is largely constrained by the ocean bathymetry which is unaffected by climate change, a further easterly distribution is constrained by temperatures which still are below the levels preferred by cod (Eide 2014). It is reasonable to expect this to be the case also for other benthic species in the Barents Sea, while pelagic species are less constrained in their spatial distributions.

The *SinMod* simulation based on the A1B climate scenario suggests a significant environmental shift in the mid 2030ies, causing a corresponding increase in the environmental carrying capacity for the NEA cod stock of about 10 %. The shift also leads to a significant increase in the cod stock biomass, most visible at medium exploitation rates and low smartness levels. In open access, the increased carrying capacity level is not fully utilised due to higher fishing effort and extended seasons. Also at low exploitation levels, the environmental effect is less visible since the cod stock already has reached a high stock level.

Previous conclusions suggesting that fisheries management decisions to have a greater impact on the development of fisheries than climate change (Eide 2007) seem to hold also after including the spatial dimension. Technological and other changes captured with the smartness parameter also have great importance and both management regimes and smartness levels clearly affect profits and fleet diversities. Given sound combinations of management and smartness levels, the climate change impacts on the NEA cod fishery could, however, significantly enhance the economic utilisation of this natural resource.

## Electronic supplementary material

Below is the link to the electronic supplementary material.

Supplementary material 1 (PDF 542 kb)

## Footnotes

1
http://www.imr.no/fishexchange/fishexchangedatabase/nb-no.

2 doi: 10.18710/B8VW6H.
