# Supplementary material for: Climate change, fisheries management and fishing aptitude affecting spatial and temporal distributions of the Barents Sea cod fishery
Source: Ambio. 2017 Oct 26;46(Suppl 3):387–99. doi: 10.1007/s13280-017-0955-1 (PMC5673871; doi:10.1007/s13280-017-0955-1)
Supplement: Supplementary file 1 — Supplementary material 1 (PDF 542 kb) [file 13280_2017_955_MOESM1_ESM.pdf]

Ambio

Supplementary Material:

Title: Climate change, fisheries management and fishing aptitude affecting spatial and temporal distributions of the Barents Sea cod fishery

Author: Arne Eide

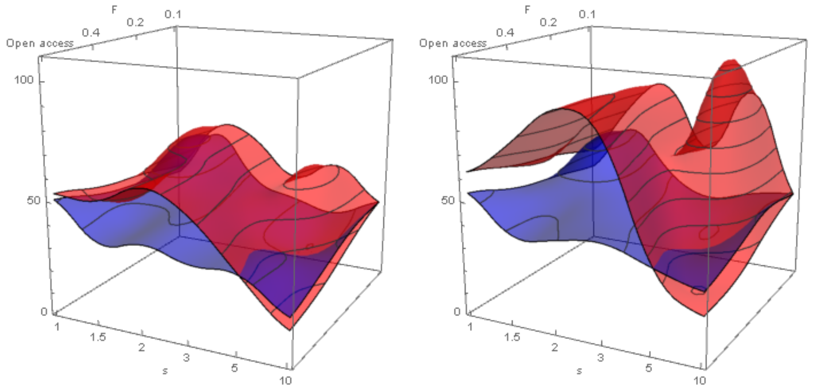

Zero scenario, all years

	F = 0.1	F = 0.2	F = 0.4	Open access
s = 1	36.50	40.20	43.80	50.90
s = 1.5	58.20	61.30	37.10	35.30
s = 2	38.10	42.80	41.40	37.90
s = 3	30.80	32.70	34.80	33.20
s = 5	26.50	19.90	30.80	32.70
s = 10	34.80	33.20	26.50	19.90

Zero scenario, last 25 years

	F = 0.1	F = 0.2	F = 0.4	Open access
s = 1	39.40	43.10	46.50	53.70
s = 1.5	60.60	63.10	43.40	38.50
s = 2	41.90	48.90	45.10	39.90
s = 3	31.50	35.70	38.80	41.90
s = 5	35.90	30.20	31.50	35.70
s = 10	38.80	41.90	35.90	30.20

A1B scenario, all years

	F = 0.1	F = 0.2	F = 0.4	Open access
s = 1	39.50	44.00	47.10	53.40
s = 1.5	61.80	64.80	46.50	53.30
s = 2	57.80	59.00	61.40	61.50
s = 3	41.80	44.40	47.70	52.50
s = 5	49.80	54.40	30.80	32.70
s = 10	34.80	28.30	19.70	14.70

A1B scenario, last 25 years

	F = 0.1	F = 0.2	F = 0.4	Open access
s = 1	47.90	53.40	56.90	62.70
s = 1.5	72.20	74.10	65.20	76.70
s = 2	83.10	86.70	91.70	92.50
s = 3	56.00	60.90	67.80	82.90
s = 5	84.40	100.00	31.40	35.70
s = 10	38.80	32.20	23.00	20.40

Figure S1: The tables show the relative sizes of total profits in the 24 simulation results obtained in each of the zero and the A1B scenario when including all simulation years (left) and the last 25 years (right). The surface plots above give a graphical representation of the two tables below each plot, where the blue surface represents the zero scenario and the red surface represents the A1B scenario.

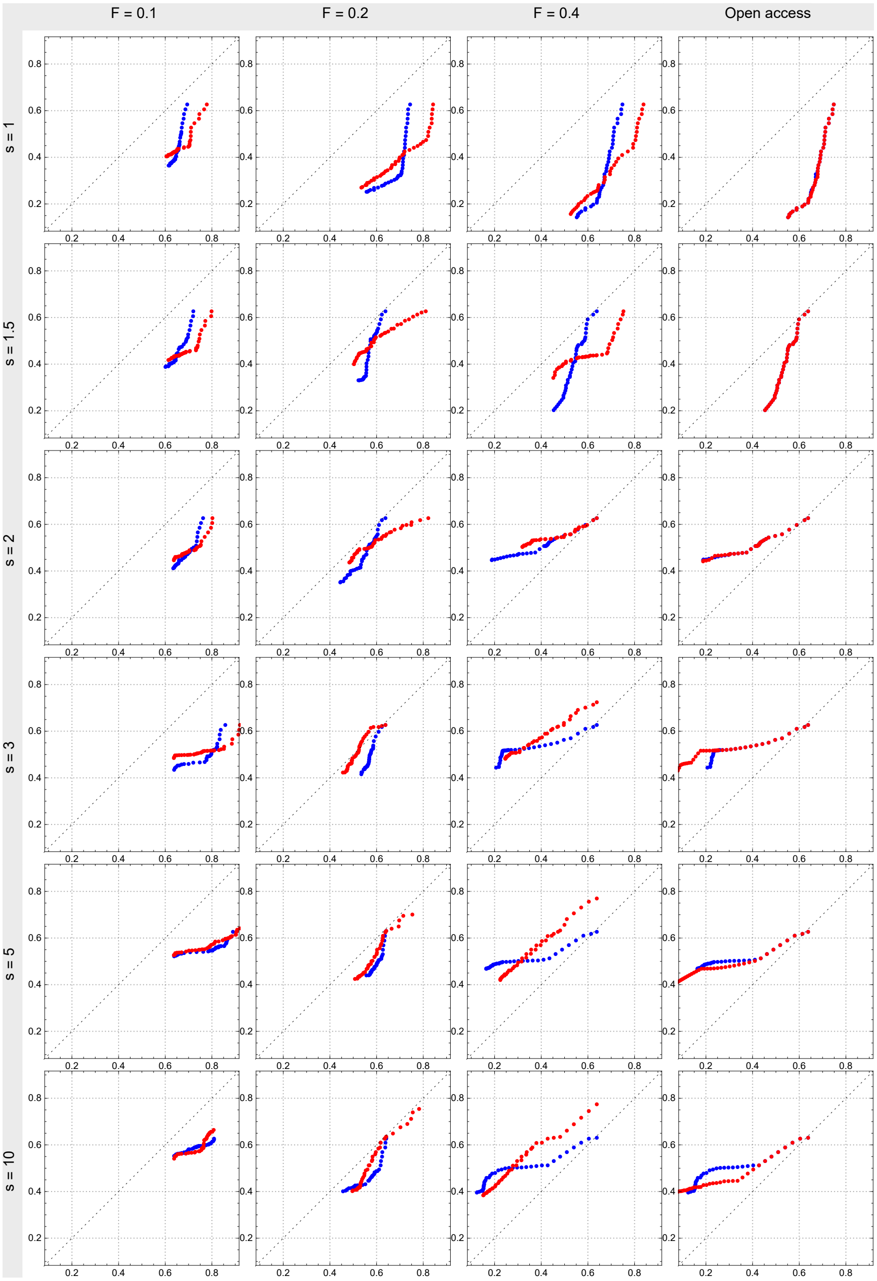

Figure S2: Fleet diversity of high sea vessels (vertical axes) versus coastal vessels (horizontal axes) in four different management regimes and six different smartness assumptions. The blue curves represent the zero scenario while the red curves show the corresponding results in the A1B scenario.

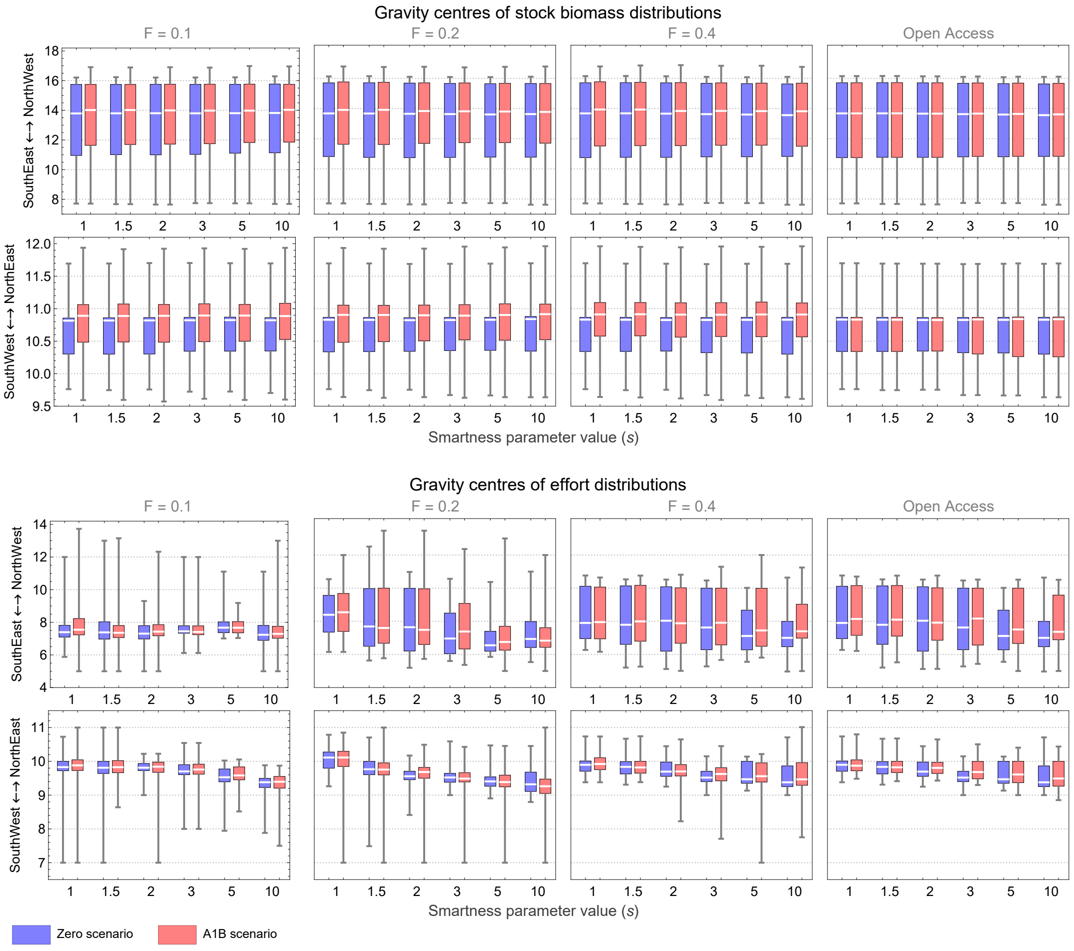

Figure S3: Gravity centres of stock biomass (above) and fishing effort (below) in the simulated NEA cod fisher 2012-2057. The top rows in the two cases (biomass and effort) represent vertical distribution while the bottom rows give the horizontal distribution in the model lattice for the two scenarios.

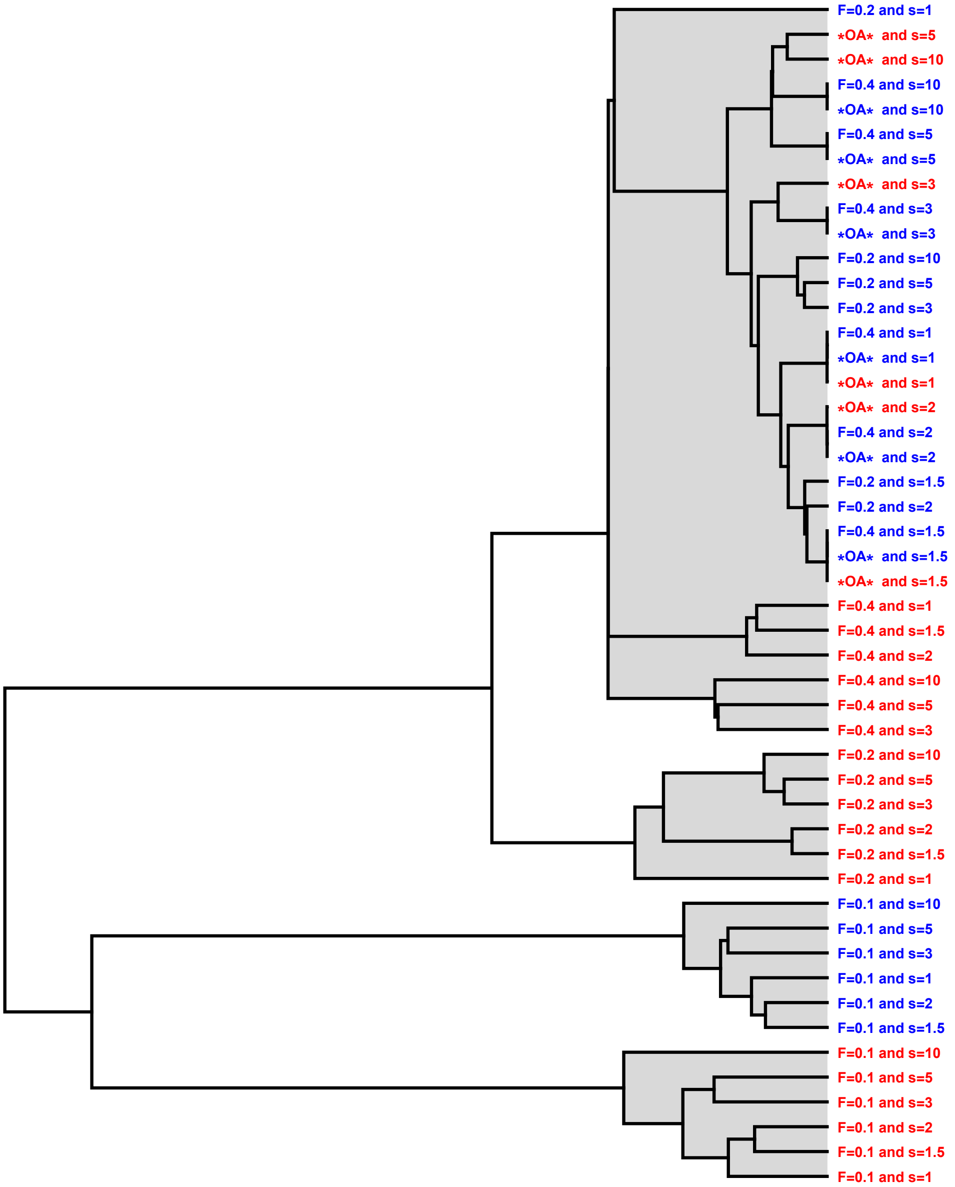

Figure S4: Dendrogram plot showing clustering patterns of spatial and temporal distribution of NEA cod stock biomasses in the performed simulation covering the time period 2012-2057. The zero scenario simulations are marked with blue labels while the A1B scenario simulation have red labels. The 24 performed simulations within each scenario are defined by different combinations of exploitation and smartness levels. The horizontal axis measures agglomerative hierarchical clustering levels in terms of squared Euclidian differences. Clustering level four is highlighted.

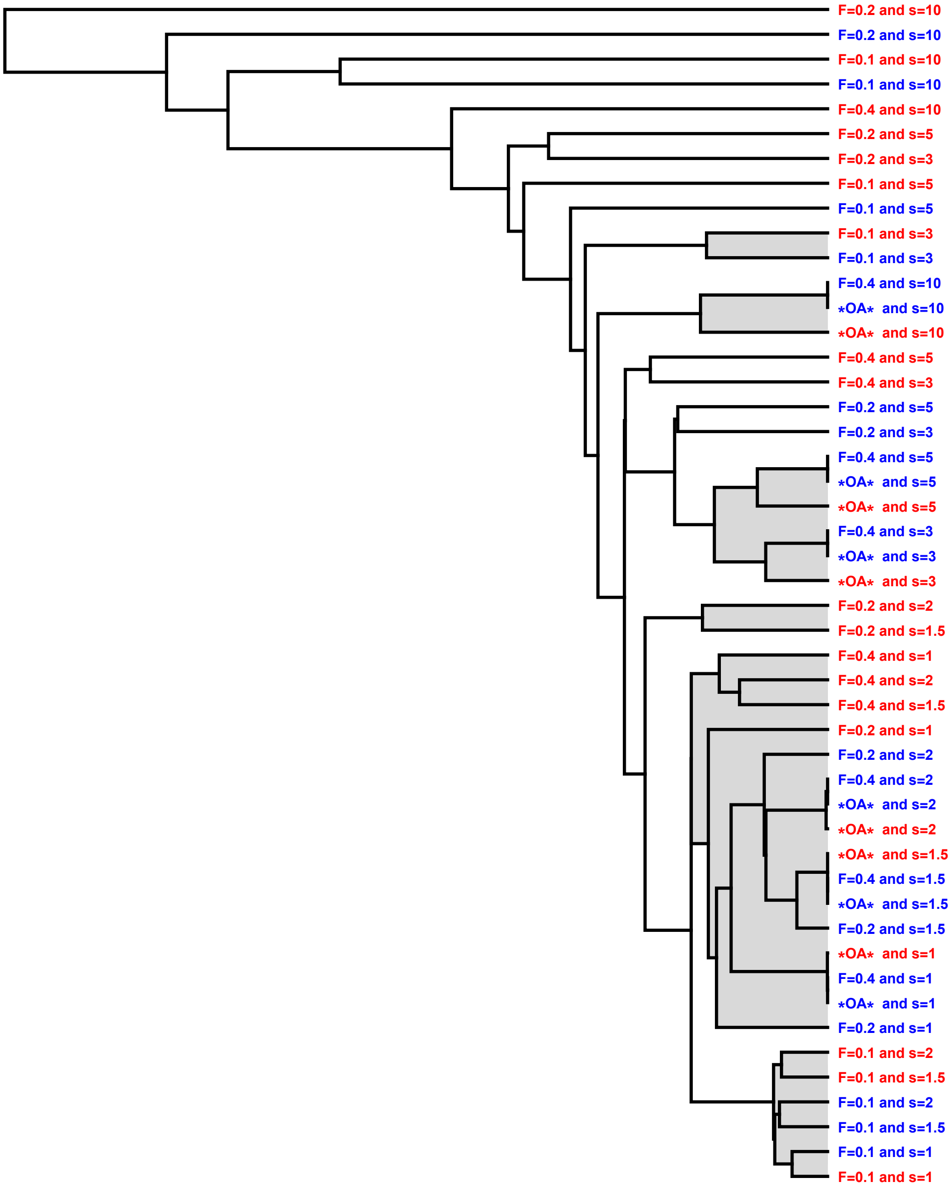

Figure S5: Dendrogram plot showing clustering patterns of spatial and temporal distribution of NEA cod stock catches in the performed simulation covering the time period 2012-2057. The zero scenario simulations are marked with blue labels while the A1B scenario simulation have red labels. The 24 performed simulations within each scenario are defined by different combinations of exploitation and smartness levels. The horizontal axis measures agglomerative hierarchical clustering levels in terms of squared Euclidian differences. Clustering level 19 is highlighted.

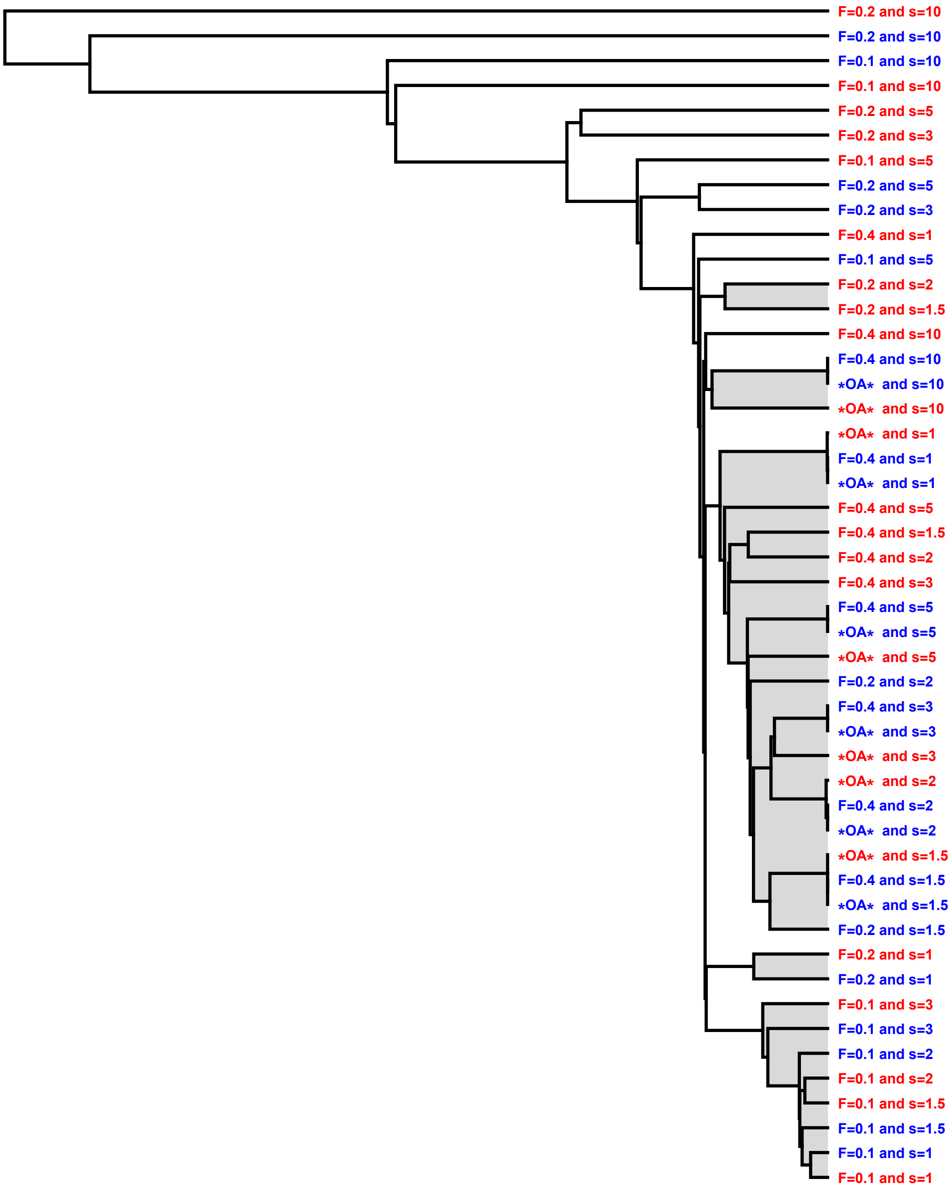

Figure S6: Dendrogram plot showing clustering patterns of spatial and temporal distribution of fishing effort in the performed simulation covering the time period 2012-2057. The zero scenario simulations are marked with blue labels while the A1B scenario simulation have red labels. The 24 performed simulations within each scenario are defined by different combinations of exploitation and smartness levels. The horizontal axis measures agglomerative hierarchical clustering levels in terms of squared Euclidian differences. Clustering level 17 is highlighted.
